# Glomus tumor of uncertain malignant potential arising in the bronchus

**DOI:** 10.1186/1749-8090-8-146

**Published:** 2013-06-07

**Authors:** Ya-Zhen Zhu, Wei-Ping Li, Zhi-Yuan Wang, Hai-Feng Yang, Qing-Lian He, Hong-Guang Zhu, Guang-Juan Zheng

**Affiliations:** 1 Department of Pathology, Guangdong provincial hospital of TCM, Guangzhou University of Chinese Medicine, 111 Dade Road, Guangzhou 510120, China; 2 Department of Pathology, Shanghai Medical College, Fudan University, Shanghai, China; 3 Guangdong Provincial Academy of Chinese Medical Sciences, Guangzhou, China

**Keywords:** Glomus tumor, Uncertain malignant potential, Bronchus

## Abstract

Glomus tumor is usually a small, benign tumor and typically occurs in the dermis or subcutis or soft tissue of the extremities and rarely in the visceral locations. Its occurrence in the main bronchus is extremely rare. The current case reported a 30-year-old woman with dyspnea on exertion and hemoptysis, she had a glomus tumor which has large size, deep location and exhibits an infiltrative margin as well as increased atypical mitotic figures. These characteristics suggest malignant behavior. However, there is little data regarding glomus tumors arising in the bronchus, the need for caution in diagnosing this case as a malignant glomus tumor must be highlighted. Therefore, the diagnosis of bronchial glomus tumor of uncertain malignant potential was favored. To the best of our knowledge, both the type and the location of this glomus tumor are extremely rare. Accumulation of more cases are needed to clarify their diagnosis and significance since there is little data regarding glomus tumors arising in the bronchus.

## Background

Glomus tumor is usually a small, benign tumor and typically occurs in the dermis or subcutis or soft tissue of the extremities and rarely in the visceral locations. Glomus tumors in the trachea, bronchi, and lung overall are sufficiently uncommon that they are not tabulated in the World Health Organization classification of lung tumors [1]. To date, only a few more than 20 cases of tracheobronchial glomus tumors have been reported [2,3] among the total, and most of them are benign. We describe an additional case of this rare entity and this case differs from the published cases in being a glomus tumor of uncertain malignant potential.

## Case presentation

**Figure 1:**
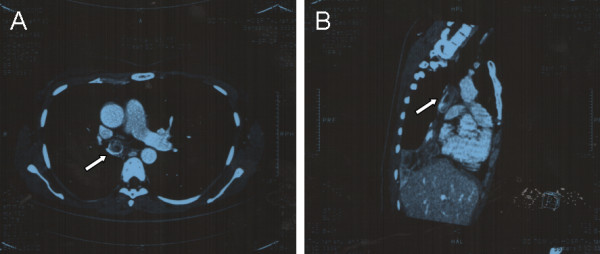
Chest computed tomography showed an intraluminal tumor shadow on the bifurcation of the right main bronchus, involving to the carina, blocking about 90% of the right main bronchus lumen (A, Cross section; B, Sagittal plane).

A 30-year-old Chinese waitress presented to our hospital complaining of polypnea on exertion for over one year, worsening for 20 days, and hemoptysis for 5 days. Her past medical history was unremarkable.The auscultation revealed attenuated respiratory sound in the right lung, no rales or wheezing was noted. A chest X-ray showed a mass in the right hilum of the lung with right lower lobe atelectasis. A CT scan revealed a mass on the bifurcation of the right main bronchus, involving to the carina, blocking about 90% of the right main bronchus lumen, with right lower lobe atelectasis and right upper lobe compensatory emphysema (Figure 1). To make a definite diagnosis of the bronchial tumor, a bronchoscopic study was performed. Bronchoscopy revealed an irregularly protruding lesion immediately on the bifurcation of the right main bronchus, which obstructed about 90% of the lumen. Biopsy tissue diagnosis showed atypical hyperplasia, and cytology did not indicate a significant number of tumorous cells. Ventilation/perfusion lung scan showed perfusion defects and ventilation defects of the right lung, while the ventilation and the perfusion of the left lung is normal.

The patient appeared aggravating polypnea, orthopnea and perspiration suddenly on the 5th day in hospital. Arterial blood gas analysis on room air breathing showed a PO2 of 105.7 mm Hg, a PCO2 of 44.4 mm Hg, and a pH of 7.37, and oxygen saturation was 94.2%. after regular treatment, the tumor was carefully removed by open surgery. Under general anaesthesia, the bronchotomy plus mass extirpation in right thoracotomy was performed. During the bronchotomy, the grey-brown friable tumor was observed, and the tumor located about 1.5 cm distal to the carina, it measured about 4.0 cm × 0.5 cm × 0.5 cm with a broad base. After the operation, the patient was transferred to the intensive care unit, and the postoperative period was uneventful, the patient went out of the hospital after 18 days.

### Pathological findings

**Figure 2:**
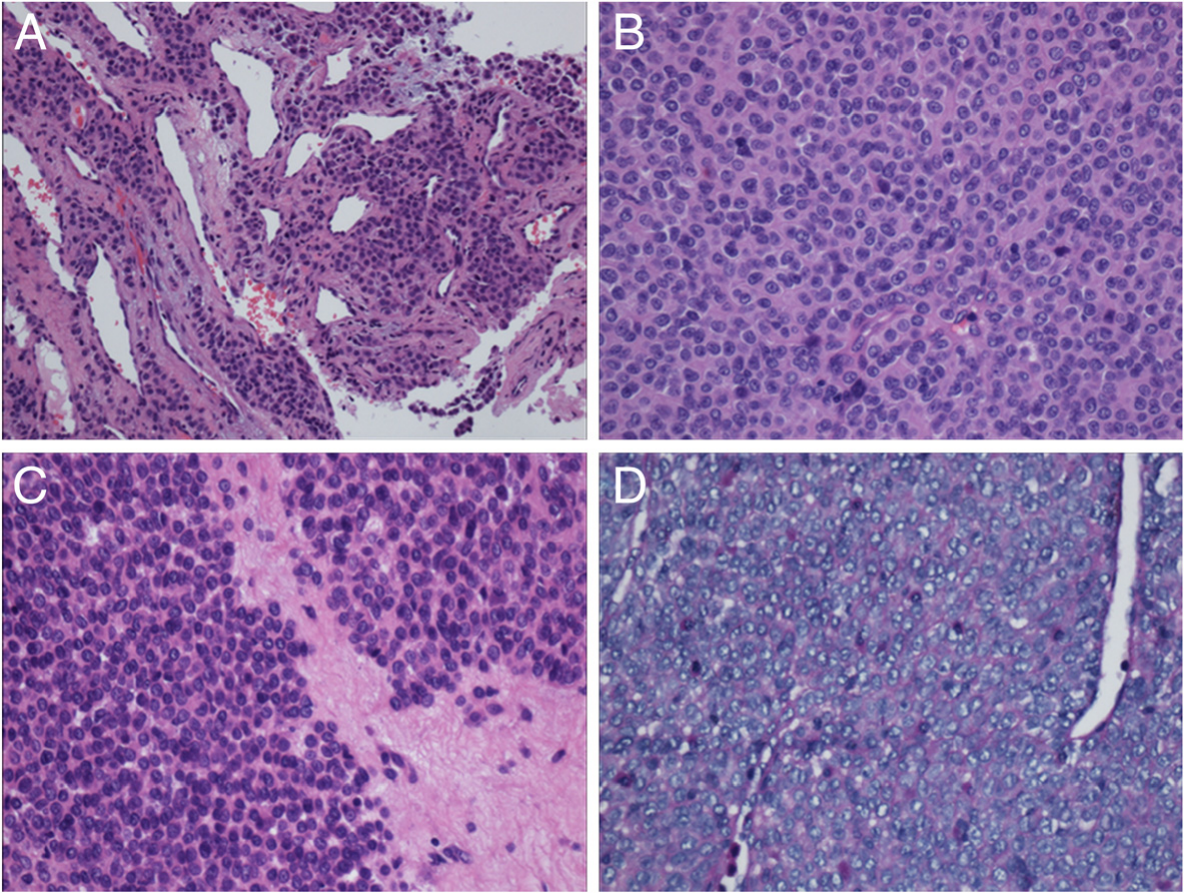
**The tumor was composed of relatively uniform round cells with perivascular aggregation, surrounding branching thin-walled vessels (A, ×200, HE staining).** The mesenchyma of the tumor showed mucinous degeneration and hyalinization (**C**, ×400, HE staining). The tumor cells appeared round nuclei, pale eosinophilic cytoplasm (**B**, ×400, HE staining) and sharply defined cell borders, which was enhanced by PAS staining (**D**, ×400). Cytological atypia and increased mitotic activity were observed locally (**B**, ×400, HE staining). HE, hematoxylin and eosin; PAS, periodic acid-Schiff.

Microscopically, the tumor was composed of relatively uniform round cells with perivascular aggregation, surrounding branching thin-walled vessels. The mesenchyma of the tumor showed mucinous degeneration and hyalinization. The tumor cells are composed of round nuclei, pale eosinophilic cytoplasm and sharply defined cell borders, which was enhanced by PAS staining. Slight cytological atypia was observed locally, and increased mitotic activity (the mitotic count was at 12-19 per 50 HPF) (Figure 2). The tumor revealed locally infiltrative growth pattern into all layers of the bronchus wall, and the surgical resection margin was involved by glomus tumor tissue. No metastasis was noted in the 6 lymph nodes below the carina.

**Figure 3:**
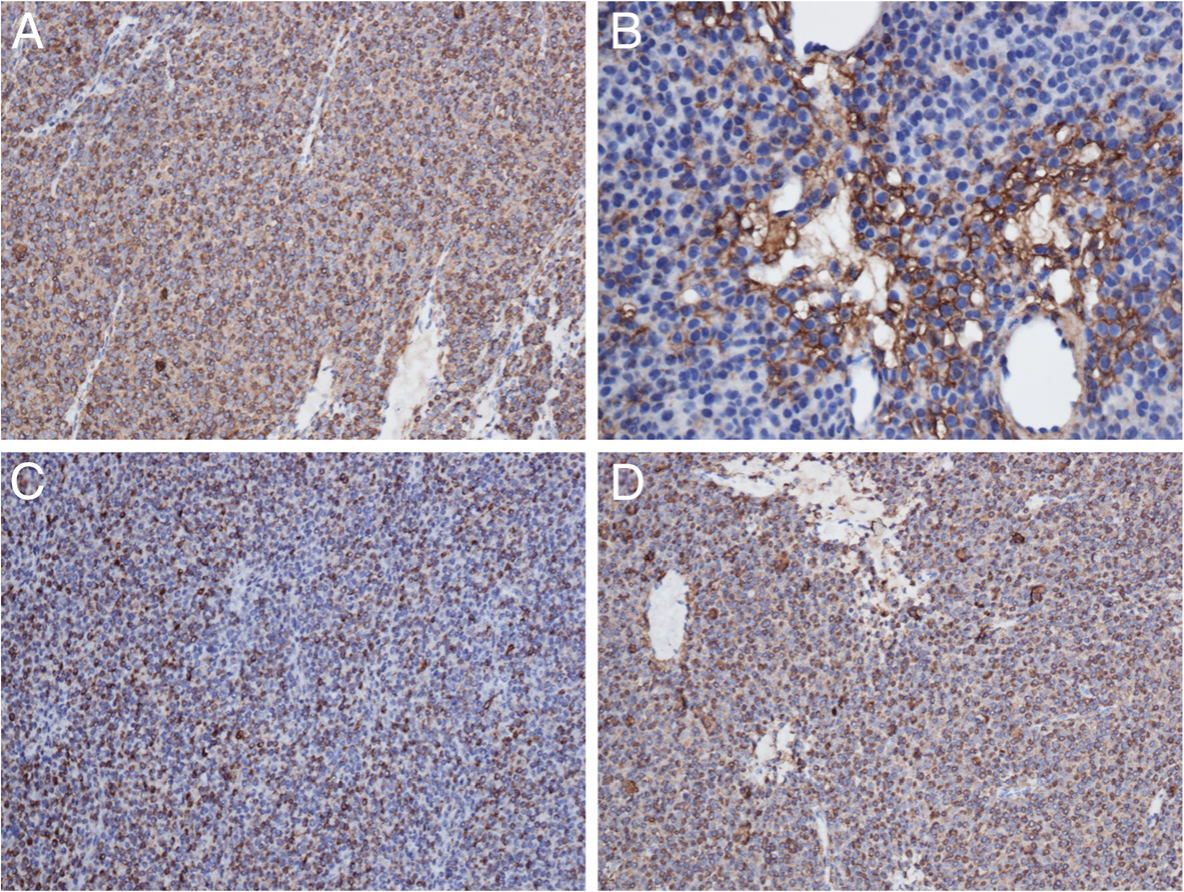
**Immunohistochemistry showed that the tumor cells were diffusely positive for SMA (A, ×200), desmin (C, ×200) and Caldesmon (D, ×200); locally positive for type IV collagen (B, ×400).** SMA, smooth muscle actin.

The tumor cells were diffusely positive for SMA, Caldesmon, desmin and vimentin; locally positive for type IV collagen. Immunostains for CD34, LCA, CD3, CD20, CK, CD56, synaptophysin, EMA, TTF1 and MPO were negative. The Ki-67 proliferative index was at least 8% (Figure 3).

The pathological features meet the criteria of malignant glomus tumor based on the criteria used for skin and soft tissue, however, there is little data regarding glomus tumors arising in the bronchus, the need for caution in diagnosing this case as a malignant glomus tumor must be highlighted. Therefore, the diagnosis of bronchial glomus tumor of uncertain malignant potential was favored, close clinical follow-up was required. It is now about 2 years since the operation, and a recent examination showed no evidence of recurrence.

## Discussion

The current case reported an extremely rare glomus tumor that was classified as glomus tumor of uncertain malignant potential despite the rare location in the bronchus. We describe an additional case of this rare entity and this case differs from the published cases in being a glomus tumor of uncertain malignant potential.The published cases of glomus tumor arising in the bronchus were reviewed and summarized in Table 1.

**Table 1:** Clinical features of the cases of bronchial glomus tumor described in the literature

**Reference**	**Age, Gender**	**Symptoms**	**Localization**	**Follow up**	**Recurrence**
Takahashi, 2006	67, male	cough	Right superior bronchial trunk	8 months	none
Altinok, 2006	83, female	hemoptysis, dyspnea, cough	the upper third of the trachea	1 year	none
Akata 2008	39, male	cough	left main bronchus	6 years	none
Kenan E. Haver, 2008	10, female	dyspnea with exertion, chest pain	middle portion of the trachea	9 months	none
Inaba 2010	67, male	hemoptysis, cough	truncus intermedius	1 year	none
Yan Shang, 2010	59, male	dyspnea, cough, chest pain	Lower Tracheal Segment	1 year	none
Nakajima, 2010	30, male	hemosputum	right truncus intermedius	10 months	none
Akira Mogi, 2011	56, female	Cough, dyspnea	the lower trachea	9 months	none
Ravenna F 2011	79, male	cough , bloody sputum	left main bronchus	5 years	none
Lang-Lazdunski, 2012	62, male	hemoptysis	Left main bronchus	5 years	none

The criteria for malignancy of glomus tumors are tumors with a deep location and a size more than 2 cm or atypical mitotic figures or marked nuclear atypia and 5 or more mitotic figures/50 HPF [4]. In this criteria, the key points indicating malignancy were “large size”, “deep location”, “atypical mitotic figure”, and “nuclear atypia”, but “infiltrative growth pattern” was considered to be unimportant [5]. The current case showed all of the above five key points. However, only locally Infiltration but no distant metastasis was present in our case, and the nuclear atypia was not so marked, therefore, the careful diagnosis of bronchial glomus tumor of uncertain malignant potential was favored.

The diagnosis of bronchial glomus tumors can be elusive. It is necessary to differentiate from hemangiopericytomas and carcinoid tumors especially for glomus tumors in unusual locations. Immunostains for SMA expression and pericellular type IV collagen expression confirm the diagnosis of glomus tumor.

Complete resection is the basic procedure for treatment of glomus tumor. In the current case, no metastasis was noted in the 6 lymph nodes below the carina and there was no evidence of distal metastasis, but the surgical resection margin was involved by glomus tumor. Therefore, close clinical follow-up is still required after surgical treatment, although the recent examination showed no evidence of recurrence after about 2 years since the operation.

## Conclusion

In summary, the current case reported a glomus tumor of uncertain malignant potential arising in the bronchus, both the type and the location of this glomus tumor are extremely rare. There is little data regarding glomus tumors arising in the bronchus, therefore, accumulation of more cases are needed to clarify its diagnosis and significance.

## Consent

Written informed consent was obtained from the patient for publication of this case report and any accompanying images. A copy of the written consent is available for review by the Editor-in-Chief of this journal.

## Abbreviations

CT: Computed tomography; PAS: Periodic acid-Schiff; HPF: High-power fields; SMA: Smooth muscle actin; LCA: Leukocyte common antigen; CK: Cytokeratin; EMA: Epithelial membrane antigen; TTF1: Thyroid transcriptor factor 1; MPO: Myeloperoxidase.

## Competing interests

The authors declare that they have no competing interests.

## Authors’ contributions

Y-ZZ was a major contributor in writing the manuscript. Q-LH carried out the immunohistochemistry. W-PL, Z-YW and H-FY collected the clinical and pathological data, H-GZ and G-JZ conceived of the study, and participated in diagnosis and helped to draft the manuscript. All authors read and approved the final manuscript.
